# Effects of daily consumption of the probiotic *Bifidobacterium animalis* subsp*. lactis* CECT 8145 on anthropometric adiposity biomarkers in abdominally obese subjects: a randomized controlled trial

**DOI:** 10.1038/s41366-018-0220-0

**Published:** 2018-09-27

**Authors:** Anna Pedret, Rosa M. Valls, Lorena Calderón-Pérez, Elisabet Llauradó, Judit Companys, Laura Pla-Pagà, Ana Moragas, Francisco Martín-Luján, Yolanda Ortega, Montse Giralt, Antoni Caimari, Empar Chenoll, Salvador Genovés, Patricia Martorell, Francisco M. Codoñer, Daniel Ramón, Lluís Arola, Rosa Solà

**Affiliations:** 1 Eurecat, Centre Tecnològic de Catalunya, Unitat de Nutrició i Salut, Reus, Spain; 2 Universitat Rovira i Virgili, Facultat de Medicina i Ciències de la Salut, Functional Nutrition, Oxidation, and Cardiovascular Diseases Group (NFOC-Salut), Reus, Spain; 3 Hospital Universitari Sant Joan de Reus, Reus, Spain; 4 Departament de Medicina i Cirurgia, Universitat Rovira i Virgili, Reus, Spain; 5 Institut Universitari d’Investigació en Atenció Primària-IDIAP Jordi Gol, Tarragona, Spain; 6 Primary Care Centre Jaume I, Institut Català de la Salut, Tarragona, Spain; 7 Primary Care Centre El Morell, Institut Català de la Salut, Tarragona, Spain; 8 Primary Care Centre Salou, Institut Català de la Salut, Tarragona, Spain; 9 Biopolis SL/Archer Daniels Midland. R&D Department (ADM Nutrition). C/Catedrático Agustín Escardino Benlloch 9, 46980-Paterna Valencia, Spain; 10 Lifesequencing/Archer Daniels Midland. R&D Department (ADM Nutrition). C/Catedrático Agustín Escardino Benlloch 9, 46980-Paterna Valencia, Spain; 11 Universitat Rovira i Virgili, Facultat de Química, Grup de Recerca en Nutrigenòmica, Tarragona, Spain

**Keywords:** Obesity, Risk factors

## Abstract

**Background:**

The effects of probiotic *Bifidobacterium animalis* subsp*. lactis* CECT 8145 (Ba8145) and those of its heat-killed form (h-k Ba8145) on human anthropometric adiposity biomarkers are unknown.

**Objective:**

To assess the effect of Ba8145 and h-k Ba8145 ingestion on anthropometric adiposity biomarkers.

**Design:**

Randomized, parallel, double-blind, placebo-controlled trial with abdominally obese individuals. Participants (*n* = 135) consumed 1 capsule/day containing 10^10^ colony forming unit (CFU) of Ba8145, 10^10^ CFU of h-k Ba8145, or placebo (maltodextrin) for 3 months.

**Results:**

Ba8145 ingestion decreased waist circumference, waist circumference/height ratio, and Conicity index (*P* < 0.05) versus its baseline. Changes versus the placebo group reached significance (*P* < 0.05) after the h-k Ba8145 treatment. Ba8145 decreased the body mass index compared with baseline and placebo group (*P* < 0.05). The decrease in visceral fat area after Ba8145 treatments reached significance (*P* < 0.05) only after h-k Ba8145. When analyses by gender were performed, significance remained only for women. Diastolic blood pressure and HOMA index decreased (*P* < 0.05) after h-k Ba8145. Gut microbiome analyses showed an increase in *Akkermansia* spp. after Ba8145 treatment, particularly in the live form, which was inversely related to weight (*P* = 0.003).

**Conclusions:**

In abdominally obese individuals, consumption of Ba8145, both as viable and mainly as heat-killed cells, improves anthropometric adiposity biomarkers, particularly in women. An increase in the gut *Akkermansia* genus appears as a possible mechanism involved. Our results support Ba8145 probiotic as a complementary strategy in obesity management.

## Introduction

The rising prevalence of overweight and obesity in Western countries is considered to be pandemic [1]. Recent guidelines of the American ACCE/ACE point out the relevance of tailored lifestyle interventions, such as physical activity and nutrition to tackle overweight and obesity [2]. Probiotics are recognized functional foods with protective effects against obesity and related cardiometabolic conditions [3]. There is a growing interest in knowing whether the beneficial properties of probiotics are preserved after killing the microorganisms (e.g., by heating or sonication) [4]. The effects of the probiotic *Bifidobacterium animalis* subsp*. lactis* CECT 8145 (Ba8145) and those of its heat-killed form (h-k Ba8145) on human adiposity biomarkers are unknown. Our preclinical data indicated that both forms of this bacterial strain decreased the body fat content, improved lipid profile, insulin sensitivity, and antioxidant status, and modulated satiety markers related to tryptophan metabolism [5, 6]. Consequently, we have assessed the effects of both Ba8145 and h-k Ba8145 on anthropometric adiposity and cardiovascular risk biomarkers, and on the gut microbiome in individuals with abdominal obesity.

## Methods

The isolation and growth of strain Ba8145 has been described previously [5]. The strain was inactivated by heat treatment (121 °C, 30 min). Products were administered in opaque hypromellose capsules as: i) placebo (300 mg of maltodextrin), ii) Ba8145, 100 mg of the live strain, 10^10^ colony forming unit (CFU)/capsule containing maltodextrin 200 mg, or iii) h-k Ba8145, 100 mg of heat-killed CECT 8145 strain at a concentration of 10^10^ CFU before the heat treatment/capsule containing maltodextrin 200 mg.

Abdominally obese subjects were recruited between September 2016 and March 2017. Inclusion criteria were: to be aged > 18 with a waist circumference (WC) ≥ 102 cm for men and ≥ 88 cm for women, according to the European guidelines [7]. Exclusion criteria are defined in Supplementary Item 1. Participants signed informed consent prior to participation. The study was approved by the Clinical Research Ethical Committee of the Hospital Universitari Sant Joan, Reus, Spain. The protocol and trial were in accordance with the Helsinki Declaration and Good Clinical Practice Guidelines of the International Conference of Harmonization (ICH GCP). Trial registration: Clinical-Trials.gov: number. NCT02921217.

A randomized, parallel, double-blind, placebo-controlled clinical trial was conducted with: placebo, Ba8145, and h-k Ba8145. Participants ingested 1 capsule/day with water for 3 months. The randomization allocation sequence was generated with a SAS 9.2 (Cary, NC: 83 SAS Institute Inc.) statistical software PROC PLAN. Blinding was maintained by using opaque capsules that did not differ among placebo and Ba8145 treatments. A 3-day dietary record and a Physical Activity Questionnaire Class AF [8] were administered to the participants at baseline and at the end of the study. Dietary recommendations were provided according to guidelines of the 2013 Adult Treatment Panel (ATP III). A fasting blood sample was obtained at baseline and after 6 and 12 weeks of each intervention. Samples were stored at -80 °C in the central laboratory’s Biobanc-REUS-IISPV (bancmb@grupsagessa.com) until required for batch analyses.

Abdominal visceral fat area (VFA) and subcutaneous fat area (SFA), as well as anthropometric adiposity biomarkers were measured at the beginning and at the end of the 3-month intervention periods. Anthropometric adiposity biomarkers other than VFA and SFA were also measured at a 6-week check-up. VFA and SFA were evaluated by magnetic resonance imaging (MRI), transverse body scan in one axial slice 5 cm over L5-S1 [9]. Anthropometric data were obtained with participants wearing lightweight clothing and no shoes. Waist circumference (WC) was measured at the umbilicus using a 150-cm anthropometric steel measuring tape. Body mass index (BMI) was calculated as the ratio between measured weight (kg)/and the square of height (m). The waist (WC, cm) to height (cm) ratio (WHtR) and the conicity index (CI) (WC (m)/ (0.109x square root of weight (kg)/height (m)) were calculated [10]. Systolic and diastolic blood pressure (SBP and DBP) were measured twice after 2–5 min of patient respite, seated, with 1-min interval in between, using an automatic sphygmomanometer (OMRON HEM-907; Peroxfarma, Barcelona, Spain). The mean values were used. Serum lipids and apolipoproteins, non-esterified fatty acids, glucose, and insulin concentrations were measured in serum by standardized enzymatic automated methods in an autoanalyzer (Beckman Coulter-Synchron, Galway, Ireland). C-reactive protein was determined by high-sensitivity immunoturbidimetry on an autoanalyzer (Roche Diagnostics Systems, Madrid, Spain). Leptin serum concentrations were determined by Elisa Kit (EMD Millipore Corporation, Billerica, USA). At the beginning and at the end of the study, a stool sample was taken with the Protocult™ collection device and frozen. DNA from stool samples was isolated, sequenced, and bioinformatically analyzed as previously described [11]. Sample size calculation and statistical analyses are described in Supplementary Item 2.

## Results

From the 187 subjects assessed for eligibility, finally 126 of them (93%, 43 men and 83 women) received allocated intervention in either placebo (*n* = 40), Ba8145 (*n* = 42), or h-k Ba8145 (*n* = 44) groups (see CONSORT Flowchart of the study in Supplementary Fig. 1). No differences in baseline characteristics were observed among intervention groups (Supplementary Item 3). Diet and physical activity were similar among groups, but fiber intake was greater in the h-k Ba8145 group versus the placebo group (*P* = 0.012) (Supplementary Item 4).

**Fig. 1:**
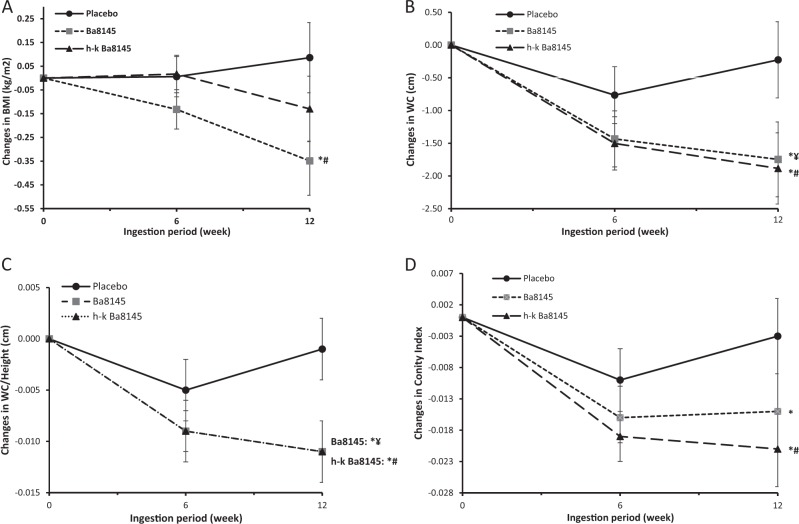
Changes in anthropometric adiposity biomarkers from baseline to the end of the study. **a** body mass index, **b** waist circumference, **c** waist circumference/height ratio, **d** conicity index. * *P* < 0.05 for intra-treatment comparisons. # *P* < 0.05 for inter-treatment comparisons (vs. Placebo). ¥ *P* < 0.07 a trend for inter-treatment comparisons (vs Placebo)

Both Ba8145 treatments decreased VFA, but significance was achieved only in the case of h-k Ba8145 (*P* < 0.05) with a borderline significance (*P* = 0.086) versus changes in the placebo group when adjusted for WC (Table 1). No changes in SFA were observed. Figure 1 shows the changes in other anthropometric adiposity biomarkers. Ba8145 treatment decreased BMI versus both its baseline and the placebo group (*P* < 0.05). Both Ba8145 interventions decreased WC, WHtR ratio, and CI (*P* < 0.05) versus its baseline. Changes versus the placebo group reached significance (*P* < 0.05) after h-k Ba8145, and a downward trend was observed after the Ba8145 treatment (*P* < 0.07) for WC and WHtR. When VFA and anthropometric adiposity data were analyzed by gender, the significance remained for women (Supplementary items 5 and 6). DBP and the HOMA-IR were reduced from baseline in the h-k Ba8145 group (*P* < 0.05). No changes were observed in SBP. No changes were observed in other biochemical measurements. Changes in leptin concentrations after both Ba8145 were not statistically significant, however they were directly related to changes in SFA (*r* = 0.367, *P* < 0.001), but not with those in VFA. No differences by gender were observed either in blood pressure or in the biochemical variables.

**Table1:** Changes in visceral fat area (VFA), measured by MRI, at 12 weeks interventions

	Intervention						Change comparisons					
	Placebo (*n* = 40)		Ba8145 (*n* = 42)		h-K Ba8145 (*n* = 44)		Ba8145 versus Placebo		h-K Ba8145 versus Placebo		h-K Ba8145 versus Ba8145	
	Post-int	Change	Post-int	Change	Post-int	Change	Mean (95%CI)	*P*	Mean (95%CI)	*P*	Mean (95%CI)	P
***Model 1***												
VFA (log), *mm* ^2^	4.12 ± 0.20	0.003 (-0.02;0.02)	4.17 ± 0.22	-0.021 (-0.04;-0.00)	4.14 ± 0.21	**-**0.022* (-0.04;-001)	-0.023 (0.05;0.01)	0.148	-0.024 (-0.05;0.01)	0.120	-0.001 (-0.03;0.03)	0.949
***Model 2***												
VFA (log), *mm*^*2*^	4.12 ± 0.20	0.004 (-0.02;0.02)	4.17 ± 0.22	-0.021 (-0.04;-0.00)	4.14 ± 0.21	-0.022* (-0.04;-002)	-0.024 (0.05;0.01)	0.120	-0.026 (-0.05;0.00)	0.086	-0.002 (-0.03;0.03)	0.907

Variables log transformed for its normalization. Data expressed as mean ± standard deviation or mean (95% Confidence Interval, CI).

Model 1, ANCOVA adjusted by age, sex, and fiber consumption at baseline.

Model 2, idem Model 1 additionally adjusted by waist circumference at baseline.

*P < 0.05

Concerning gut microbiota, the percentages at baseline for enterotype 1 were 67%, 63%, and 81% for placebo, Ba8145, and h-k Ba8145 groups, respectively. Changes after placebo and h-k Ba8145 treatments were of -2%, and -8%, whereas an increase of 21% was observed after Ba8145 treatment (Supplementary Item 7). *Akkermansia* ssp. increased 0.008%, 0.584%, and 0.053 % in placebo, Ba8145, and h-k Ba8145 groups, respectively, in an inverse relationship with weight after reverse transformation of the data (*r* = −0.035; *P* = 0.003) (Supplementary Fig. 2**)**. Median *Akkermansia* percentage content after Ba8145 treatments was 1.8% lower in participants over 90 kg versus those ≤ 90 kg (*P* = 0.043).

## Discussion

This 3-month intervention study assessed the effect of oral Ba8145 and its h-k Ba8145 form on anthropometric adiposity biomarkers and cardiovascular risk factors. Ba8145 treatments reduced VFA, BMI, WC, WHtR ratio, and CI when compared from baseline to the end of the study, particularly in women. With exception of BMI, the greatest reductions were observed after the h-k Ba8145 intervention. After h-k Ba8145 intervention, DBP and the HOMA index both decreased.

Ba8145 treatment decreased the BMI by -0.349 kg/m^2^. This reduction was similar to those obtained after 3 months of *Lactobacillus gasseri* SBT2055 (-0.5 kg/m^2^) [12], or *L. plantarum* TENSIA (-0.4 kg/m^2^) [13] after 3 weeks, and around to those obtained after probiotic consumption in a recent meta-analysis (-0.27 kg/m^2^) [6], or after 6–12 months of a multifactorial lifestyle intervention (-0.41 kg/m^2^) [14]. Average reductions in WC of -1.75 cm for Ba8145 and -1.84 cm for h-k Ba8145 were in the range to those reported after probiotic treatments [12, 15] or 6–12 months of multifactorial lifestyle interventions [14, 16]. The average changes in WHtR (-0.011) and CI (-0.018) were similar to those reported after an 8-week consumption period of a probiotic mix of *Lactobacillus* and *Bifidobacterium* (-0.01 and -0.02 for WHtR and CI, respectively) [17]. Mean VFA changes, -3.66 cm^2^ after Ba8145 and -7.01 cm^2^ for h-k Ba8145, were in the range to those reported after *Bifidobacterium lactis* GCL2505 (-6.6 cm^2^)[18] during 8 and 12 weeks or *Lactobacillus gasseri* SBT2055 (-5.6 cm^2^) [12]. Adiposity accumulation is an independent risk factor for coronary heart disease, hypertension, type-2 diabetes, and impaired glucose tolerance [3]. Accordingly, in the present study, a decrease in DBP and HOMA-IR, a surrogate marker of insulin resistance, was observed after h-k Ba8145 intervention. In our study although no differences were observed between Ba8145 treatments, the heat-killed form appears to be more effective that the live one. The fact that modified probiotics proved more efficient than living strains has been described previously [4]. Mechanisms involved have been related to the host immune system regulation [4, 19]. An increase in leptin sensitivity due to probiotic administration cannot be discarded [20] despite the fact that Ba8145 treatments failed to modify leptin concentrations, possibly due to the lack of changes in SFA, which is the main source for leptin [21].

Previous reports in animal models described a protective effect of *Akkermansia* on obesity [22]. However, data on the issue from human clinical trials are scarce [23]. In our trial, and concomitantly with the decrease in obesity biomarkers, Ba8145 treatments increased the incidence of *Akkermansia* spp. in the gut in an inverse relationship with weight decrease. Consistent with this fact, the maximum increase in *Akkermansia* spp. was observed after the Ba8145 live-form administration, when the maximum decrease in BMI occurred. Also, *Akkermansia* content was 1.8% lower in participants over 90 kg after Ba8145 treatments. The shift to enterotype 1 after Ba8145 treatment could be related with the *Bifidobacterium* genus, since it promotes growth of *Bacteroides*, the main player in enterotype 1.

The study has strengths and limitations (Supplementary Item 8). One limitation is the inability to assess potential interactions between capsules and other dietary components. Also, the fact that most of the subjects enrolled in the study were female could account for the lack of significance of changes in the male group. One strength of the study lies in the fact that the probiotic strains were administered alone. In summary, this study shows that ingestion of the probiotic *Bifidobacterium animalis* subsp. *lactis* CECT 8145, as viable cells and mainly in a heat-killed form, reduces anthropometric adiposity biomarkers in abdominally obese individuals, particularly in women. An increase in gut *Akkermansia* genus appears as a possible mechanism involved. Our results support the use of Ba8145 probiotic as a complementary strategy in the management of obesity.

## Electronic supplementary material

Supplementary Information

Supplemental Figure 1

Supplemental Figure 2
