# Supplementary material for: Effects of daily consumption of the probiotic Bifidobacterium animalis subsp. lactis CECT 8145 on anthropometric adiposity biomarkers in abdominally obese subjects: a randomized controlled trial
Source: Int J Obes (Lond). 2018 Sep 27;43(9):1863–8. doi: 10.1038/s41366-018-0220-0 (PMC6760601; doi:10.1038/s41366-018-0220-0)
Supplement: Supplementary file 1 — Supplementary Information [file 41366_2018_220_MOESM1_ESM.doc]

SUPPLEMENTARY INFORMATION (6 Items)

**Supplementary item 1. Exclusion criteria**

Exclusion criteria were:

a) WC < 102 cm for men and <88 cm for women, and > 150 cm for both gender

b) BMI ≥ 40 kg/m^2^

c) antibiotic treatment 30 days before the inclusion in study

d) diabetes (glucose ≥ 126 mg/dL or pharmacological treatment)

e) pregnancy, having given birth in the past year, planning pregnancy in the next 6 months, or lactation

f) abnormal thyroid status

g) anemia

h) alcoholism

i) following a hypocaloric diet and/or pharmacological treatment for weight loss

j) intake of food supplements or medications that could affect body weight

k) intestinal disease

l) any other conditions that were not suitable for the trial as evaluated by the physician.

**Supplementary item 2. Sample size calculation and statistical analyses**

Sample size was estimated assuming a Type I error of 0.05 (two-sided) and at least 80% power for detecting a mean area of 5 cm^2^ in a between-group reduction of the abdominal VFA. Based on this, 43 individuals were assigned to each group. The standard deviation was estimated to be 13.02 cm^2^.^1^

In statistical analyses, the normality of variables was assessed by the Kolmogorov-Smirnov test. Non-parametric variables were log transformed. We used the Kruskal-Wallis test or 1-factor analysis of variance (ANOVA) to determine differences in baseline characteristics. Analyses were made by intention-to-treat. Comparisons among treatments were carried out by an ANCOVA model adjusted by age and sex. Anthropometric adiposity measurements were additionally adjusted for fiber consumption. Statistical significance was defined as a *P* value less than 0.050 for a 2-sided test. We performed analyses by using SPSS for Windows, version 22 (IBM corp., Armonk, NY, USA). For massive genome sequencing, Wilcoxon test, linear model correlation and graphical representation was performed using R package version 3.4.4.

1.Kadooka Y, Sato M, Ogawa A, Miyoshi M, Uenishi H, Ogawa H, *et al*. Effect of Lactobacillus gasseri SBT2055 in fermented milk on abdominal adiposity in adults in a randomised controlled trial. *Br J Nutr* 2013;**110**:1696–1703.

**Supplementary Item 3.** Baseline characteristics of the participants

| Variable | Placebo  (n = 40) | Ba8145  (n= 42) | h-K Ba8145 (n=44) | *P* | |
| --- | --- | --- | --- | --- | --- |
| Age. years | 43.4 ± 9.2 | 45.8 ± 11.3 | 45.7 ± 8.8 | 0.463 | |
| Female, n | 27 | 24 | 32 | | 0.081 |
| Menopause, % | 26.7 | 20 | 15.6 | | 0.104 |
| Smoking habits, % |  |  |  | |  |
| Never | 36.6 | 29.3 | 34.1 | | 0.324 |
| Smoker | 33.3 | 28.6 | 38.1 | | 0.316 |
| Exsmoker | 41.9 | 14 | 44.1 | | 0.071 |
| SBP, mm Hg | 129 ± 16 | 123 ± 22 | 127 ± 20 | | 0.441 |
| DBP, mm Hg | 82 ± 11 | 79 ± 12 | 81 ± 11 | | 0.518 |
| BMI, kg/m^2^ | 31.7 ± 4.2 | 32.3 ± 4.4 | 31.1 ± 4.1 | | 0.407 |
| Waist circumference, cm | 105.6 ± 12.0 | 108.4 ± 10.6 | 105.3 ± 9.2 | | 0.345 |
| WC/height ratio | 0.64 ± 0.06 | 0.65 ± 0.06 | 0.64 ± 0.05 | | 0.343 |
| Conicity index | 1.34 ± 0.07 | 1.36 ± 0.06 | 1.35 ± 0.07 | | 0.423 |
| Physical activity, AU^*^ | 6.68 (4.5-8.9) | 9.07 (6.5-11.6) | 8.42(6.1-10.7) | | 0.335 |
| DData expressed as mean ± standard deviation or percentage and ^*^median(25^th^-75^th^percentile). AAbbreviations: Ba8145, alive probiotic; h-K Ba8145, heat-killed probiotic; SBP, systolic blood pressure; DBP, diastolic blood pressure; AU, arbitrary units: 0-1, inactive; 2-3, minimally active; 4-5, slightly active; 6-11, moderately active; ≥12, very active. P for ANOVA. | | | | | |

**Supplementary Item 4.** Dietary composition before and after treatments interventions.

| Variables | | Placebo (n=40) | P | Ba8145 (n=42) | P | h-k Ba8145 (n=44) | P | P Value* | |
| --- | --- | --- | --- | --- | --- | --- | --- | --- | --- |
| Energy, kcal/day | Baseline | 2109 ± 437 |  | 2105 ± 631 |  | 2062 ± 498 |  |  |  |
|  | 12 weeks | 1936 ± 603 | 0.053 | 2060 ± 614 | 0.632 | 1955 ± 677 | 0.132 | NS |  |
| HC, % energy | Baseline | 36.0 ± 6.2 |  | 37.8 ± 7.7 |  | 35.3 ± 5.7 |  |  |  |
|  | 12 weeks | 35.9 ± 7.3 | 0.302 | 33.2 ± 7.6 | <0.001 | 36.2 ± 6.6 | 0.498 | 0.002^¥^ |  |
| HC, g | Baseline | 187 ± 56 |  | 196 ± 68 |  | 179 ± 48 |  |  |  |
|  | 12 weeks | 168 ± 58 | 0.017 | 171 ± 68 | 0.004 | 174 ± 62 | 0.511 | NS |  |
| Protein, % energy | Baseline | 17.4 ± 2.8 |  | 196 ± 68 |  | 18.3 ± 3.2 |  |  |  |
|  | 12 weeks | 18.4 ± 3.7 | 0.191 | 171 ± 68 | 0.010 | 17.6 ± 2.9 | 0.276 | 0.021^¥^ |  |
| Protein, g | Baseline | 89.4 ± 18.3 |  | 88.3 ± 30.3 |  | 92.9 ± 25.1 |  |  |  |
|  | 12 weeks | 85.4 ± 21.4 | 0.252 | 93.2 ± 27.8 | 0.355 | 83.3 ± 21.8 | 0.010 | 0.035^¥^ |  |
| Total fat, % energy | Baseline | 43.6 ± 5.2 |  | 41.8 ± 6.6 |  | 44.3 ± 6.9 |  |  |  |
|  | 12 weeks | 43.8 ± 7.7 | 0.831 | 45.3 ± 5.8 | 0.001 | 43.5 ± 5.9 | 0.545 | 0.023^¥^ |  |
| Total fat, g | Baseline | 102 ± 23.8 |  | 99.4 ± 32.7 |  | 102 ± 30.2 |  |  |  |
|  | 12 weeks | 97.8 ± 41.7 | 0.523 | 105 ± 33.3 | 0.371 | 95.9 ± 33.6 | 0.201 | NS |  |
| SFA, % energy | Baseline | 12.8 ± 2.4 |  | 12.2 ± 3.0 |  | 12.7 ± 2.6 |  |  |  |
|  | 12 weeks | 12.4 ± 3.3 | 0.462 | 12.4 ± 2.6 | 0.712 | 12.7 ± 2.7 | 0.959 | NS |  |
| SFA, g | Baseline | 30.2 ± 8.9 |  | 29.3 ± 13.3 |  | 29.6 ± 9.7 |  |  |  |
|  | 12 weeks | 28.2 ± 15.1 | 0.433 | 29.0 ± 10.4 | 0.887 | 28.2 ± 11.9 | 0.354 | NS |  |
| MUFA, % energy | Baseline | 19.4 ± 3.7 |  | 19.6 ± 3.8 |  | 19.6 ± 4.1 |  |  |  |
|  | 12 weeks | 19.9 ± 4.7 | 0.400 | 21.7 ± 4.2 | 0.003 | 19.2 ± 4.5 | 0.666 | NS |  |
| MUFA, g | Baseline | 44.7 ± 11.0 |  | 45.5 ± 12.0 |  | 46.1 ± 16.7 |  |  |  |
|  | 12 weeks | 43.7 ± 18.5 | 0.725 | 48.9 ± 14.5 | 0.218 | 41.7 ± 16.3 | 0.119 | NS |  |
| PUFA, % energy | Baseline | 7.8 ± 2.4 |  | 6.8 ± 2.6 |  | 8.0 ± 2.8 |  |  |  |
|  | 12 weeks | 7.8 ± 3.1 | 0.914 | 7.3 ± 3.8 | 0.242 | 7.6 ± 3.2 | 0.551 | NS |  |
| PUFA, g | Baseline | 18.7 ± 8.0 |  | 16.7 ± 10.0 |  | 18.5 ± 8.1 |  |  |  |
|  | 12 weeks | 17.8 ± 10.3 | 0.382 | 17.7 ± 10.7 | 0.510 | 17.0 ± 9.3 | 0.320 | NS |  |
| Fiber, g/day^a^ | Baseline | 34.0 (18.1-73.8) |  | 20.5 (13.5-49.0) |  | 21.1 (13.0-39.5) |  |  |  |
|  | 12 weeks | 29.7 (15.9- 62.9) | 0.014 | 21.5 (11.2-33.2) | 0.550 | 30.9 (14.3-44.3) | 0.203 | 0.012^†^ |  |
| Alcohol, g/d^a^ | Baseline | 6.58 (1.67-18.1) |  | 2.20 (0.00-19.2) |  | 2.37 (0.00-15.3) |  |  |  |
|  | 12 weeks | 3.96 (0.08-16.5) | 0.179 | 2.87 (0.00-16.5) | 0.788 | 2.35 (0.00-17.0) | 0.534 | NS |  |
| Data expressed as mean ± standard deviation or mean (95% Confidence Interval, CI), ^a^median percentiles 25th-75th). Abbreviations: HC, carbohydrate; SFA, Saturated fatty acid; MUFA, Monounsaturated fatty acid; PUFA, Polyunsaturated fatty acid. ANCOVA Model adjusted by age and sex. (log), variable log transformed. *P<0.05; †P<0.005. Intra-intervention comparisons by Student t test or Wilcoxon test. *P value for intervention differences: ^†^ h-K Ba8145 intervention versus Placebo; ^¥^ h-K Ba8145 intervention versus Ba8145 intervention. ANOVA and Mann Whitney tests. | | | | | | | | |  |

**Supplementary Item 5.** Changes in visceral fat area (VFA) by gender, measured by MRI, at 12 weeks interventions

|  |  | | Intervention | | |  | | Change comparisons | | | | | | | | | |
| --- | --- | --- | --- | --- | --- | --- | --- | --- | --- | --- | --- | --- | --- | --- | --- | --- | --- |
|  | Placebo  (n = 40) | | Ba8145  (n= 42) | | | h-K Ba8145  (n=44) | | Ba8145  versus Placebo | | | h-K Ba8145  versus Placebo | | | | h-K Ba8145  versus Ba8145 | | |
|  | Post-int Change | | Post-int | | Change | Post-int | Change | Mean P  (95%CI) | | | Mean  (95%CI) | | P | | Mean  (95%CI) | | P |
| **Men** (n= 28) | | | | | | | | | | | | | | | | | |
| ***Model 1*** | | | | | | | | | | | | | | | | | |
| VFA (log), *mm^2^* | 4.25±0.11 | -0.030  (-0.07;0.01) | 4.35±0.12 | -0.027  (-0.06;0.01) | | 4.30±0.16 | **-**0.008  (-0.04; 003) | | 0.003  (-0.05;0.06) | 0.915 | | 0.022  (-0.03;0.08) | | 0.422 | 0.019  (-0.03;0.07) | 0.447 | |
| ***Model 2*** | | | | | | | | | | | | | | | | | |
| VFA (log), *mm^2^* | 4.25±0.11 | -0.030  (-0.07;0.02) | 4.35±0.12 | -0.026  (-0.06;0.01) | | 4.30±0.16 | -0.009  (-0.05; 003) | | 0.004  (0.05;0.01) | 0.895 | | 0.021  (-0.04;0.08) | | 0.453 | 0.017  (-0.04;0.07) | 0.503 | |
| **Women** (n=66) | |  |  |  | |  |  | |  |  | |  | |  |  |  | |
| ***Model 1*** |  |  |  |  | |  |  | |  |  | |  | |  |  |  | |
| VFA (log), *mm^2^* | 4.06±0.20 | 0.007  (-0.02;0.04) | 4.07±0.20 | -0.019  (-0.05;0.01) | | 4.07±0.15 | **-**0.027*  (-0.05; -001) | | -0.027  (-0.07;0.01) | 0.207 | | -0.034  (-0.07;0.005) | | 0.086 | -0.008  (-0.05;0.03) | 0.686 | |
| ***Model 2*** |  |  |  |  | |  |  | |  |  | |  | |  |  |  | |
| VFA (log), *mm^2^* | 4.06±0.20 | 0.008  (-0.02;0.04) | 4.07±0.20 | -0.020  (-0.05;0.01) | | 4.07±0.15 | **-**0.027*  (-0.05; -001) | | -0.028  (-0.07;0.01) | 0.197 | | - 0.035  (-0.07;0.005) | | 0.084 | -0.007  (-0.05;0.03) | 0.703 | |
| Variables log transformed for their normalization. Data expressed as mean ± standard deviation or mean (95% Confidence Interval, CI). at baseline. Model 1, ANCOVA adjusted by age and fiber consumption at baseline. Model 2, idem Model 1 additionally adjusted by waist circumference at baseline. **P*< 0.05 | | | | | | | | | | | | | | | | | |

**Supplementary Item 6. Changes in anthropometric adiposity measures by gender after 12 weeks of intervention**

|  |  | | Intervention | |  | | Change comparisons | | | | | |
| --- | --- | --- | --- | --- | --- | --- | --- | --- | --- | --- | --- | --- |
| Variable | Placebo  (n = 40) | | Ba8145  (n= 42) | | h-K Ba8145  (n=44) | | Ba8145  versus Placebo | | h-K Ba8145  versus Placebo | | h-K Ba8145  versus Ba8145 | |
|  | Post-int | Change | Post-int | Change | Post-int | Change | Mean  (95%CI) | P | Mean  (95%CI) | P | Mean  (95%CI) | P |
| **Men** |  |  |  |  |  |  |  |  |  |  |  |  |
| BMI, *kg/m^2^* | 33.1 ± 3.4 | -0.189  (-0.82;0.44) | 31.5 ± 3.6 | **-**0.059  (-0.59; 0.47) | 31.1 ±3.1 | -0.191  (-0.74;0.35) | 0.130  (-0.72;0.98) | 0.755 | -0.002  (-0.85;0.85) | 0.996 | -0.132  (-0.85;0.85) | 0.723 |
| WC, *cm* | 111 ± 7.8 | -0.141  (-3.63;1.35) | 110 ± 9.2 | -0.744  (-2.83;1.35**)** | 110 ± 8.2 | -1.482  (-3.64;0.68) | 0.397  (-3.15;0.11) | 0.810 | -0.341  (-3.70;3.02) | 0.837 | -0.738  (-3.73;2.26) | 0.617 |
| WHtR | 0.64 ± 0.04 | -0.006  (-0.02;0.00) | 0.63 ± 0.06 | -0.004  (-0.02;0.007) | 0.63 ± 0.05 | -0.008  (-0.02;0.004) | 0.002  (-0.02;0.02) | 0.822 | -0.002  (-0.02;0.02) | 0.839 | -0.004  (-0.02;0.01) | 0.632 |
| CI | 1.35 ± 0.04 | -0.006  (-0.03;0.02) | 1.36 ±0.06 | -0.008  (-0.03;0.01) | 1.37 ± 0.06 | -0.013  (-0.03;0.007) | -0.002  (-0.03;0.03) | 0.891 | -0.007  (-0.04;0.02) | 0.633 | -0.005  (-0.03;0.02) | 0.702 |
| **Women** |  |  |  |  |  |  |  |  |  |  |  |  |
| BMI, *kg/m^2^* | 30.8 ± 4.4 | 0.085  (-0.25;0.42) | 31.3 ± 4.6 | **-**0.456^*^  (-0.80; -0.11) | 31.2 ± 4.4 | -0.057  (-0.37;0.25) | -0.541  (-1.0;-0.05) | 0.032 | -0.141  (-0.61;0.33) | 0.554 | 0.400  (-0.06;0.86) | 0.087 |
| WC *cm* | 101 ± 12.9 | -0.151  (-1.53;1.23) | 102 ± 10.2 | -2.149^*^  (-3.56;-0.74**)** | 102 ± 8.5 | -1.962^*^  (-3.24;-0.69) | -1.998  (-4.01;0.01) | 0.052 | -1.812  (-3.75;0.12) | 0.066 | 0.187  (-1.69;2.06) | 0.843 |
| WHtR | 0.62 ± 0.07 | -0.001  (-0.009;0.00) | 0.64 ± 0.06 | -0.013^*^  (-0.02;-0.005) | 0.62 ± 0.05 | -0.012^*^  (-0.02;-0.004) | -0.012  (-0.02;0.00) | 0.053 | -0.011  (-0.02;0.01) | 0.073 | 0.001  (-0.01;0.01) | 0.814 |
| CI | 1.31 ± 0.07 | -0.004  (-0.02;0.01) | 1.33 ±0.07 | -0.018^*^  (-0.03;-0.003) | 1.32 ± 0.06 | -0.024^*^  (-0.04;-0.01) | -0.014  (-0.04;0.008) | 0.209 | -0.020  (-0.04;0.002) | 0.069 | -0.006  (-0.03;0.01) | 0.588 |
| BMI, body mass index; WC, waist circumference; WHtR , waist (WC, cm) to height(cm) ratio; CI, Conicity index (WC (m)/ (0.109x square root of weight (kg)/height (m))  Data expressed as mean (95% confidence interval, CI). ANCOVA model adjusted by age and fiber consumption at baseline. ***.***^*^P<0.05 | | | | | | | | | | | | |

**Supplementary item 7.** Changes in gut microbiota after interventions: Percentage of subjects allocated in enterotype 1 and median percentage of *Akkermansia spp* .

| Treatment | Pre-intervention | | Post-intervention | |
| --- | --- | --- | --- | --- |
|  | Enterotype 1  (%) | *Akkermansia genus* median %  (interquartile range*)* | Enterotype 1  (%) | *Akkermansia genus* median %  (interquartile range) |
| Placebo | 67 | 0.014 (0.008-0.659) | 65 | 0.022 (0.005-0.367) |
| Ba8145 | 63 | 0.053 (0.008-1.426) | 81 | 0.637 (0.008-3.294) |
| h-k Ba8145 | 82 | 0.505 (0.007-2.441) | 74 | 0.558 (0.014-2.695) |

**Supplementary item 8.** Limitations and strengths of the study

This study presents some limitations such as:

a) the inability to assess potential interactions between the capsules and other diet components

b) participants were obese subjects and this fact could impair the extrapolation of the results to the general population

c) additional or different effects that would have been observed over longer periods is unknown

As strengths:

1. in most of the clinical trials the probiotic strain has been used as a functional ingredient on a food matrix (i.e. yogurt) or in combination with a prebiotic fiber in a symbiotic product. Therefore, it is difficult to determine if the effect is due to the probiotic strain or to the other components in the final product. In our clinical trial both Ba8145 treatments are studied alone and the detected effects are exclusively due to the Ba8145 strain.
2. the fact that the heat-killed cells of Ba8145 are functional pave the way for the use of this probiotic strain in a large variety of food matrices, including those that suffer a thermal treatment of sterilization (i.e. fruit juices, bakery products).
