# Supplementary figures and images for: Effects of daily consumption of the probiotic Bifidobacterium animalis subsp. lactis CECT 8145 on anthropometric adiposity biomarkers in abdominally obese subjects: a randomized controlled trial
Source: Int J Obes (Lond). 2018 Sep 27;43(9):1863–8. doi: 10.1038/s41366-018-0220-0 (PMC6760601; doi:10.1038/s41366-018-0220-0)

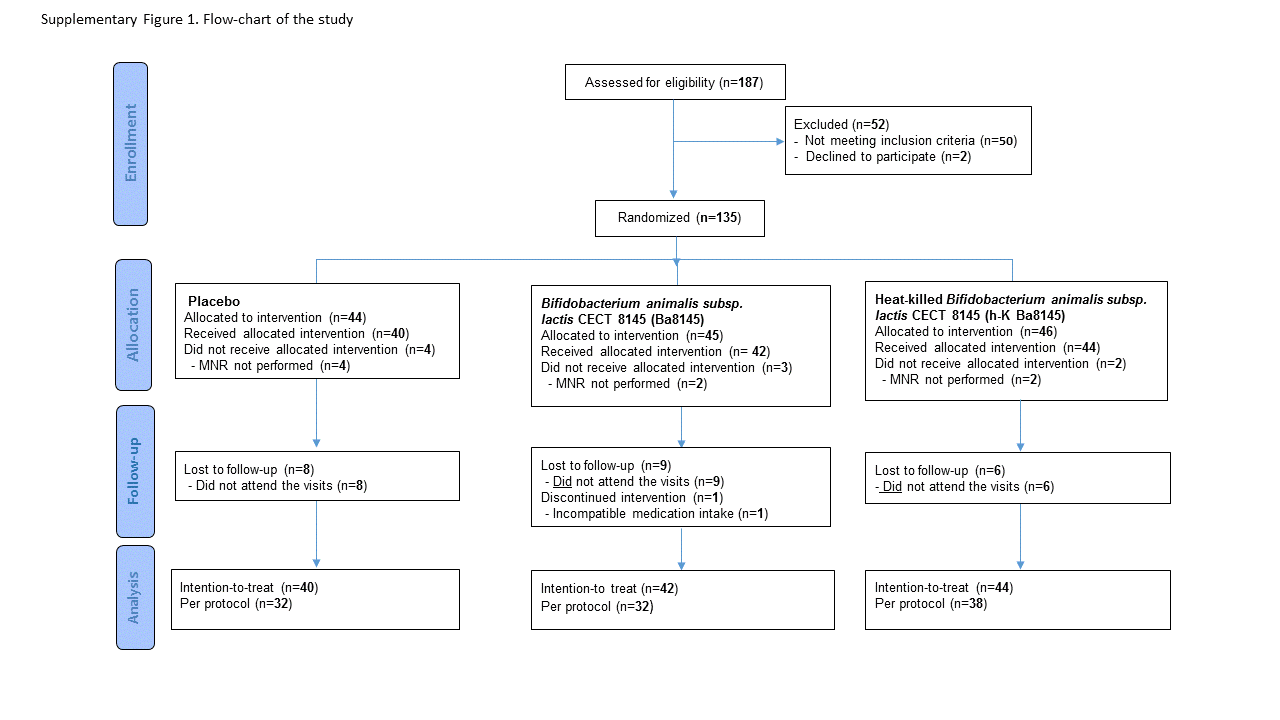

Supplement: Supplementary file 2 — Supplemental Figure 1 [file 41366_2018_220_MOESM2_ESM.gif]

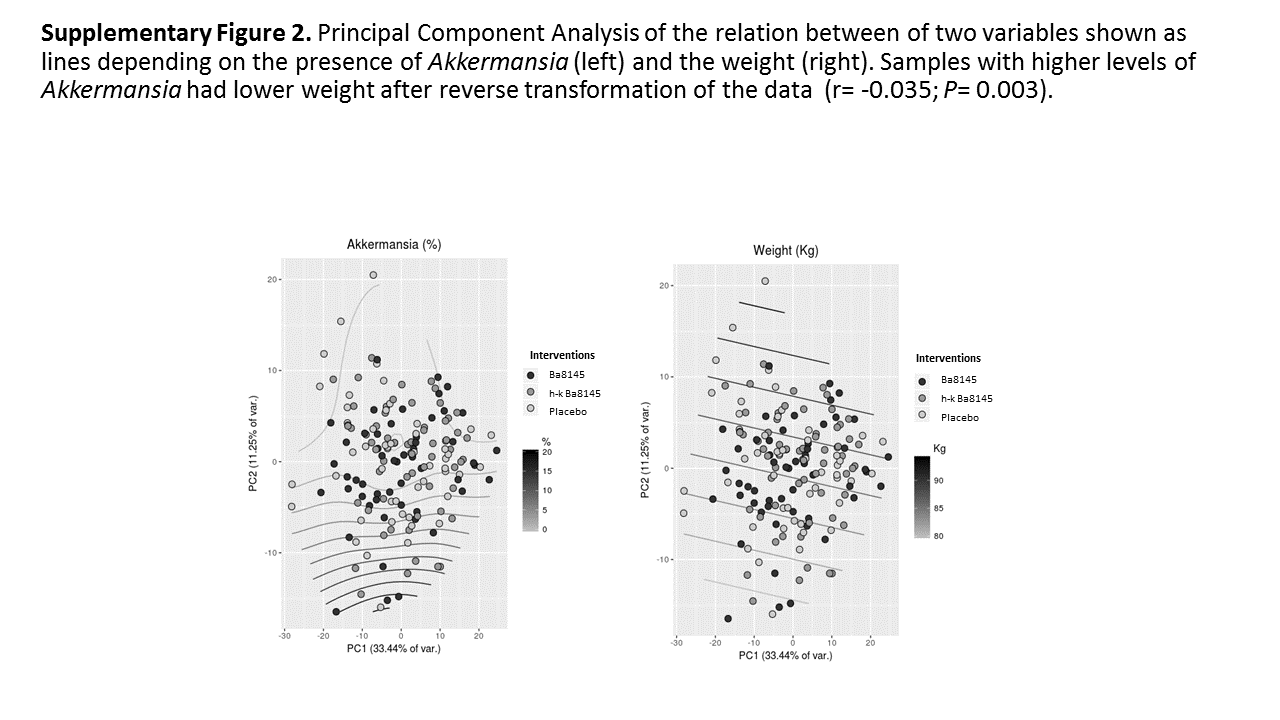

Supplement: Supplementary file 3 — Supplemental Figure 2 [file 41366_2018_220_MOESM3_ESM.gif]
